# Synchronous Duodenal Carcinoid and Adenocarcinoma of the Colon

**DOI:** 10.4021/wjon554w

**Published:** 2012-10-28

**Authors:** Limin Gao, Seth Lipka, Jorge Hurtado-Cordovi, Boris Avezbakiyev, Alejandro Zuretti, Kaleem Rizvon, Paul Mustacchia

**Affiliations:** a Department of Internal Medicine, Associated with North Shore- Long Island Jewish Health Care System, Nassau University Medical Center 2201 Hempstead Turnpike, East Meadow NY 11554, USA; b Department of Internal Medicine, division of Hematology/Oncology, Associated with North Shore- Long Island Jewish Health Care System. Nassau University Medical Center 2201 Hempstead Turnpike, East Meadow NY 11554, USA; c Department of Pathology, Associated with North Shore- Long Island Jewish Health Care System, Nassau University Medical Center 2201 Hempstead Turnpike, East Meadow NY 11554, USA; d Department of Internal Medicine, division of Gastroenterology, Associated with North Shore- Long Island Jewish Health Care System, Nassau University Medical Center 2201 Hempstead Turnpike, East Meadow NY 11554, USA

**Keywords:** Synchronous tumor, Duodenal carcinoid

## Abstract

Carcinoid tumors are a histological subtype of well differentiated, low to intermediate grade, slow-growing neuroendocrine malignancies capable of secreting bioactive peptides, such as 5-hydroxytryptamine (5-HT, serotonin), chromogranin-A and chromogranin-C. Here we present a case of a duodenal carcinoid that simultaneously occurred with adenocarcinoma of the colon. A 59-year-old male with a past medical history of hepatitis C and hypertension presented complaining of worsening abdominal pain associated with 2 - 3 episodes per week of bright red blood per rectum for the past month. He also reported a 20 pounds weight loss in the last 6 months. Social history was significant for a 15 pack year history. Vitals on admission were within normal limits. Physical exam was significant for right upper quadrant tenderness without guarding, rebound, or organomegaly. Rectal exam revealed no blood or masses. Laboratory results showed iron deficiency anemia with hemoglobin of 9.6 K/mm^3^. Esophagogastroduodenoscopy revealed a 4 mm duodenal polyp. Colonoscopy was terminated early secondary to a large circumferential obstructing mass found in the descending colon. Immunohistochemistry of the duodenal biopsy was positive for synaptophysin and chromogranin-A; consistent with the diagnosis of stage I carcinoid tumor. Biopsy results of the colonic mass showed a stage I well-differentiated adenocarcinoma. The patient underwent a left colectomy and partial duodenectomy; he remains in remission after 2 year of close follow up. When the diagnosis of small bowel carcinoid is made, further screening for other primary neoplasms should be sought to prevent potential late stage diagnosis of synchronous malignancies. This is crucial because patients’ demise usually result from the associate tumor and not the carcinoid component. Finally, we would like to raise clinician’s awareness regarding the incidence of this entity since some of the studies suggest that it is more common than it was previously thought.

## Introduction

Carcinoid tumors are relatively slow-growing neuroendocrine tumors that are capable of secreting bioactive peptides, such as 5-hydroxytryptamine (5-HT, serotonin), chromogranin-A and chromogranin-C. The most common site for carcinoid tumor origin is the gastrointestinal tract (73-85%) with the majority of patients being asymptomatic [1]. While the occurrence of duodenal carcinoid is only 1.5-5%, its association with synchronous colon adenocarcinoma is rare. We described a case of a duodenal carcinoid that simultaneously occurred with adenocarcinoma of the colon [1-2].

## Case Report

**Figure 1:**
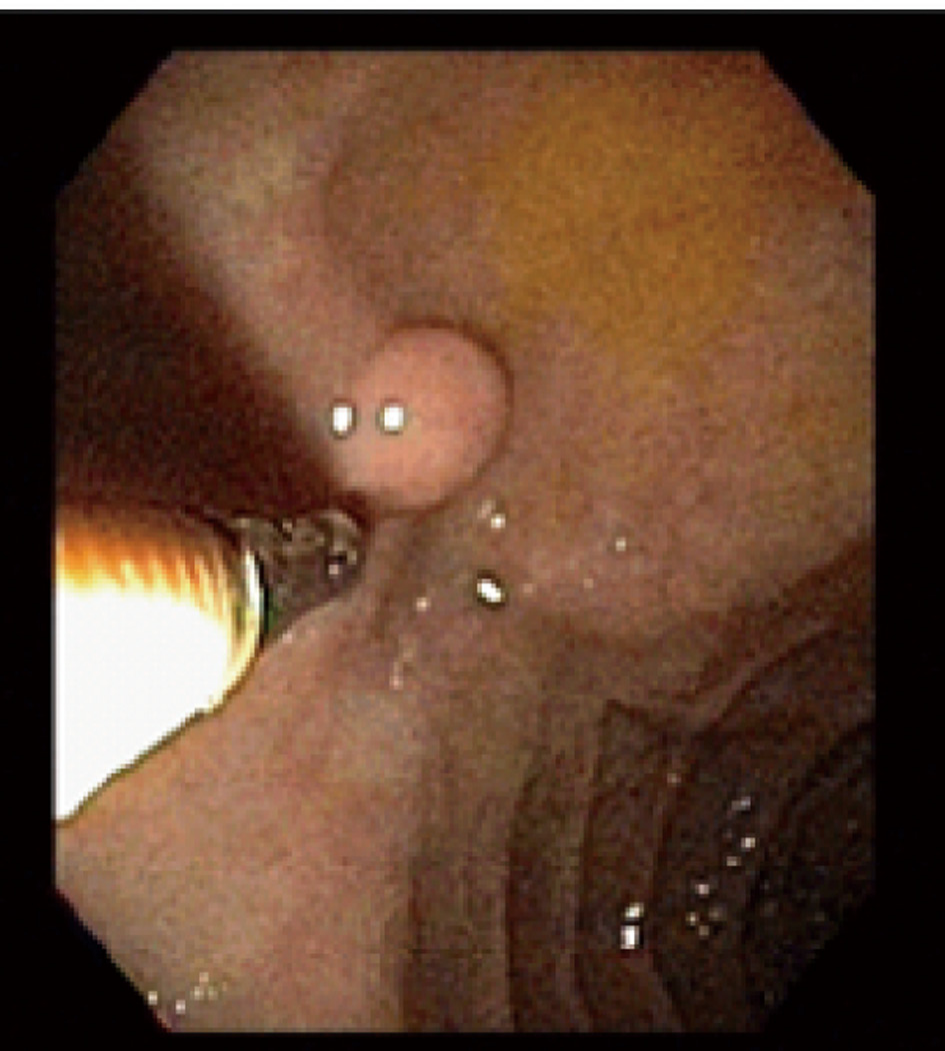
A 4 mm duodenal polyp found during endoscopy.

**Figure 2:**
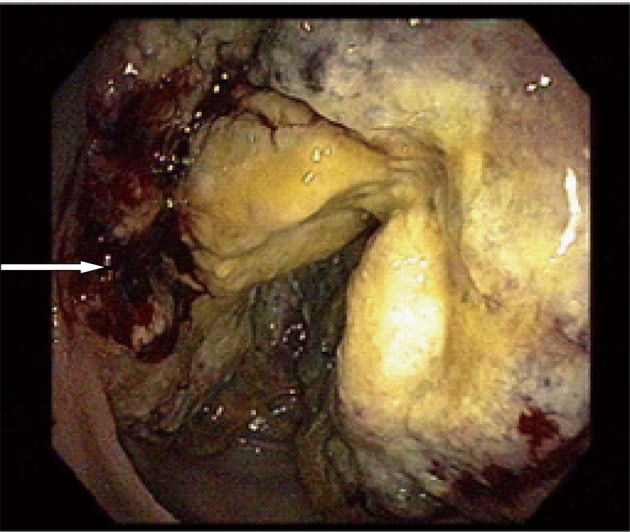
A large circumferential obstructing mass located 40 cm away from the anal verge with areas of hemorrhages (arrow).

**Figure 3:**
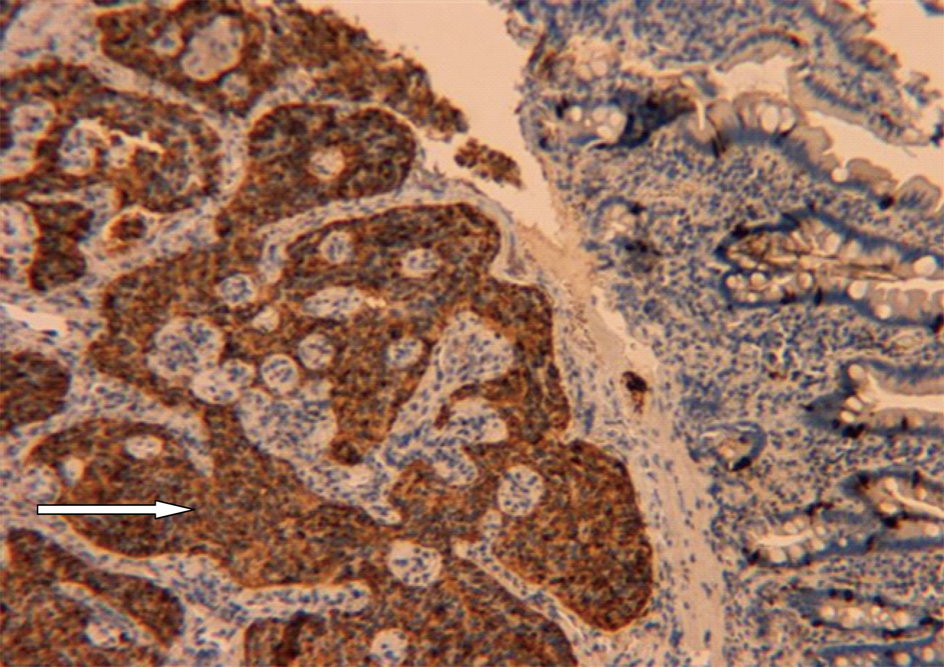
Immunohistochemistry stain positive for chromogranin-A (arrow), suggesting the diagnosis of carcinoid malignancy (200 ×).

**Figure 4:**
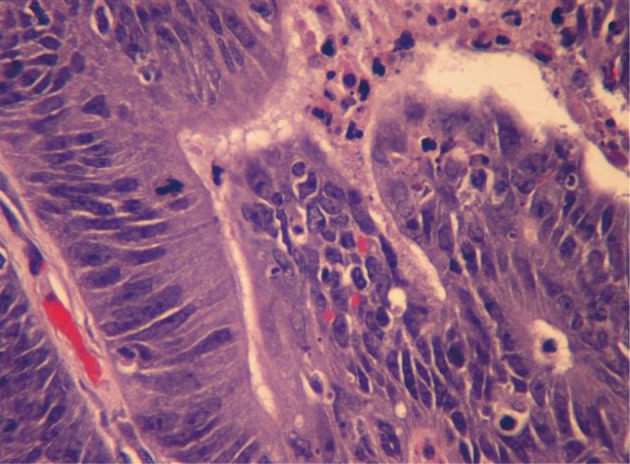
H&E stain of the descending colon biopsy showing atypical cells with pleomorphic nucleus suggesting the diagnosis of adenocarcinoma (400 ×).

A 59-year-old male with a past medical history of hepatitis C and hypertension presented complaining of worsening abdominal pain associated with bright red blood per rectum for the past month. The pain was 7/10 in intensity, constant, non-radiating, with no alleviating or aggravating factors. He admits having 2 - 3 episodes of bloody stools per week. He also reported a 20 pounds weight loss in the last 6 months. Review of systems was otherwise negative. Social history was significant for a 15 pack year history. He denied family history gastrointestinal malignancies. Vitals on admission were within normal limits. Physical exam was significant for right upper quadrant tenderness without guarding, rebound, or organomegaly. Rectal exam revealed no blood or masses. Laboratory results showed iron deficiency anemia with hemoglobin of 9.6 K/mm^3^. Liver related tests and coagulation profile were within normal limits. Esophagogastroduodenoscopy revealed a 4mm duodenal polyp (Fig. 1). Colonoscopy was terminated early secondary to a large circumferential obstructing mass (Fig. 2) found in the descending colon. Immunohistochemistry of the duodenal biopsy was positive for synaptophysin and chromogranin-A (Fig. 3); consistent with the diagnosis of stage I carcinoid tumor. Biopsy results of the colonic mass showed a stage I well-differentiated adenocarcinoma (Fig. 4). The patient underwent a left colectomy and partial duodenectomy. He remains in remission after 2 year of close follow up.

## Discussion

In the gastrointestinal tract, carcinoid tumors are found most often in the jejunum(13.4-17.6%), rectum(10-18.5%), colon(7.6-9.5%) and appendix(2.4-7.6%); with duodenal carcinoids accounting for less than 2% of all carcinoids [2, 3]. The annual incidence of duodenal carcinoids is 0.07/100,000 [4]. Carcinoid tumors can present with a variety of clinical symptoms and are difficult to diagnose. However, most carcinoids have an asymptomatic, indolent course with late metastases. Furthermore, 80% of cases present with the classic carcinoid syndrome symptoms, likely because up 91% are found to have distant metastases at the time of diagnosis. The liver is the most common site of metastasis. These symptoms are due to increased production of 5-hydroxytryptamine (5-HT), and include flushing, diarrhea, palpitations, spasmodic abdominal pain and bronchial constriction. Diagnosis depends on histopathological examination and positive reactions to neuroendocrine markers such as synaptophysin, neuron-specific enolase, and glycoprotein chromogranin A. The rapid technological advancements such as endoscopic procedures, ultrasound(US), computed tomography(CT), magnetic resonance imaging(MRI), positron emission tomography(PET), and radio labeled somatostatin receptor scintigraphy(SRS, OctreoScan), have enhanced significantly the diagnosis of carcinoid tumors allowing for a more accurate delineation of metastases. The five year survival dependent on the stage and location of the tumor; with only a 25% survival for patients with metastasis, compared to an 88% for those with local disease [5, 6].

Multiple factors must be taken into consideration when planning therapy. Well-differentiated, nonfunctional duodenal carcinoids that are limited to the mucosa/submucosa and are no more than 10 mm in size can be endoscopically removed. In these cases the preferred procedure to achieve complete excision of the tumor is endoscopic mucosal resection (EMR). Larger masses (10 - 20 mm) with similar characteristics as above can be removed by endoscopy or surgery. Surgical management is the treatment of choice for duodenal carcinoids that are nonfunctional but more than 20 mm in size. Tumors extending beyond the submucosa (T2-T4) or that have metastasized to lymph nodes should be managed surgically [7-9].

The incidence of synchronous tumors of the colon ranges from 2 to 11 percent [10]. While adenocarcinomas are the most common colorectal malignancy, the incidence of carcinoid malignant tumors is 1-4/1,000,000 per year [11]. According to the SEER (Surveillance, Epidemiology, and End Results Program, National Cancer Institute, USA), 29% of patients with gastrointestinal carcinoid had an additional malignancy. Gerstle et al studied 69 patients with gastrointestinal tract carcinoids and found that 29 of them (42%) had a synchronous tumor, with colorectal adenocarcinoma being the most common associate malignancy. Berge and Linell reported that out of 199 patients with carcinoid tumor 81 (40.7%) had one or more coexisting synchronous malignancy with up to 35.8% of the accompanying neoplasm occurring in the gastrointestinal tract. Most of the patients with synchronous malignancies had no symptoms relating to the carcinoid tumor. Asymptomatic duodenal carcinoids are usually small and confine to the submucosa; therefore, it is possible to perform a segmental intestinal resection as it was done in our case. Whenever a synchronous tumor with a non-metastatic carcinoid component is encounter, its prognosis is determined by the associate malignancy, and the vast majority of patients succumb to the non-carcinoid constituent [12-16].

In conclusion, when the diagnosis of small bowel carcinoid is made, further screening for other primary neoplasms should be sought to prevent potential late stage diagnosis of synchronous malignancies. This is crucial because patients’ demise usually result from the associate tumor and not the carcinoid component. Finally, we would like to raise clinician’s awareness regarding the incidence of this entity since some of the studies suggest that it is more common than it was previously thought.
